# Genetically modified organisms: adapting regulatory frameworks for evolving genome editing technologies

**DOI:** 10.1186/s40659-022-00399-x

**Published:** 2022-10-20

**Authors:** Pablo Rozas, Eduardo I. Kessi-Pérez, Claudio Martínez

**Affiliations:** 1 Institute for Biological and Medical Engineering, Schools of Engineering, Medicine and Biological Sciences, Pontificia Universidad Católica de Chile, Santiago, Chile; 2 Centro de Estudios en Ciencia y Tecnología de Alimentos (CECTA), Universidad de Santiago de Chile (USACH), Santiago, Chile; 3 Departamento de Ciencia y Tecnología de los Alimentos, Universidad de Santiago de Chile (USACH), Santiago, Chile

**Keywords:** Biomedicine, Climate change, Food, Genetically modified organism (GMO), New breeding techniques (NBT), Regulatory frameworks, Transgenesis

## Abstract

Genetic modification of living organisms has been a prosperous activity for research and development of agricultural, industrial and biomedical applications. Three decades have passed since the first genetically modified products, obtained by transgenesis, become available to the market. The regulatory frameworks across the world have not been able to keep up to date with new technologies, monitoring and safety concerns. New genome editing techniques are opening new avenues to genetic modification development and uses, putting pressure on these frameworks. Here we discuss the implications of definitions of living/genetically modified organisms, the evolving genome editing tools to obtain them and how the regulatory frameworks around the world have taken these technologies into account, with a focus on agricultural crops. Finally, we expand this review beyond commercial crops to address living modified organism uses in food industry, biomedical applications and climate change-oriented solutions.

## Background

Genetic modification of living organisms for food, feed, industrial, medical, and environmental uses has been an intense field of research and economic interest since the development of modern agriculture. From the development of DNA recombination in the 70’s, the rapid and transversal implementation of genetic engineering impacted several industries such as medicine, food, feed and scientific research itself. Nevertheless, the idea of modification of living organisms is older than DNA recombination technology.

Throughout history, humanity has tried to improve yields, resources optimization, nutritional content, and organoleptic characteristics of plant crops through various plant improvement techniques. *i.e.*, plant breeding. These techniques include artificial selection, selective crosses, mutagenesis induced by chemical or physical agents, and genetic engineering, among others [1, 2]. In this context, genetic engineering has contributed to accelerate the developing times of new plant varieties and increasing their diversity, capacities and applications.

One of the most widely used genetic engineering technique, and a pioneer in the field of agricultural biotechnology, is transgenesis, which consists of the transfer of genetic material from one organism to another of a different species. This process makes it possible to achieve certain traits of technological, productive, nutritional, or research interest. The most frequently developed commercial traits are resistance to pathogens, tolerance to abiotic stress, and resistance to herbicides [3–6].

Although the potential of transgenesis in the agricultural development, the definition of a genetically modified organism (GMO) has been a controversial topic for consumers and an evolving concept in the literature and regulatory frameworks since the first applications of transgenesis became commercially available in the 1990s. Despite being associated with this technique, current international efforts have led to a broader definition of “living modified organism” (LMO) written down into The Cartagena Protocol on Biosafety to the Convention on Biological Diversity [7]. This defines a LMO as “any living organism that possesses a novel combination of genetic material obtained through the use of modern biotechnology”; where “living organism” is defined as “any biological entity capable of transferring or replicating genetic material, including sterile organisms, viruses and viroids”. On the other hand, “modern biotechnology” is defined as “the application of:

- in vitro nucleic acid techniques, including recombinant deoxyribonucleic acid (DNA) and direct injection of nucleic acid into cells or organelles, or
- fusion of cells beyond the taxonomic family, that overcome natural physiological reproductive or recombination barriers and that are not techniques used in traditional breeding and selection.”

This definition of LMO, hereinafter "GMO” for the scope of this review given its use in the historical literature, has a profound impact on regulatory strategies worldwide. In this review, we focus on the current technologies employed to develop GMOs, especially crops, and how regulatory frameworks are evolving to take new technologies into account. Moreover, we will discuss how the potential of genetically modified (GM) crops and other organisms can be exploited in other industries and in biomedical applications, as well as current efforts developed to address the challenge of climate change.

## Main text

### GMOs global landscape

**Fig. 1:**
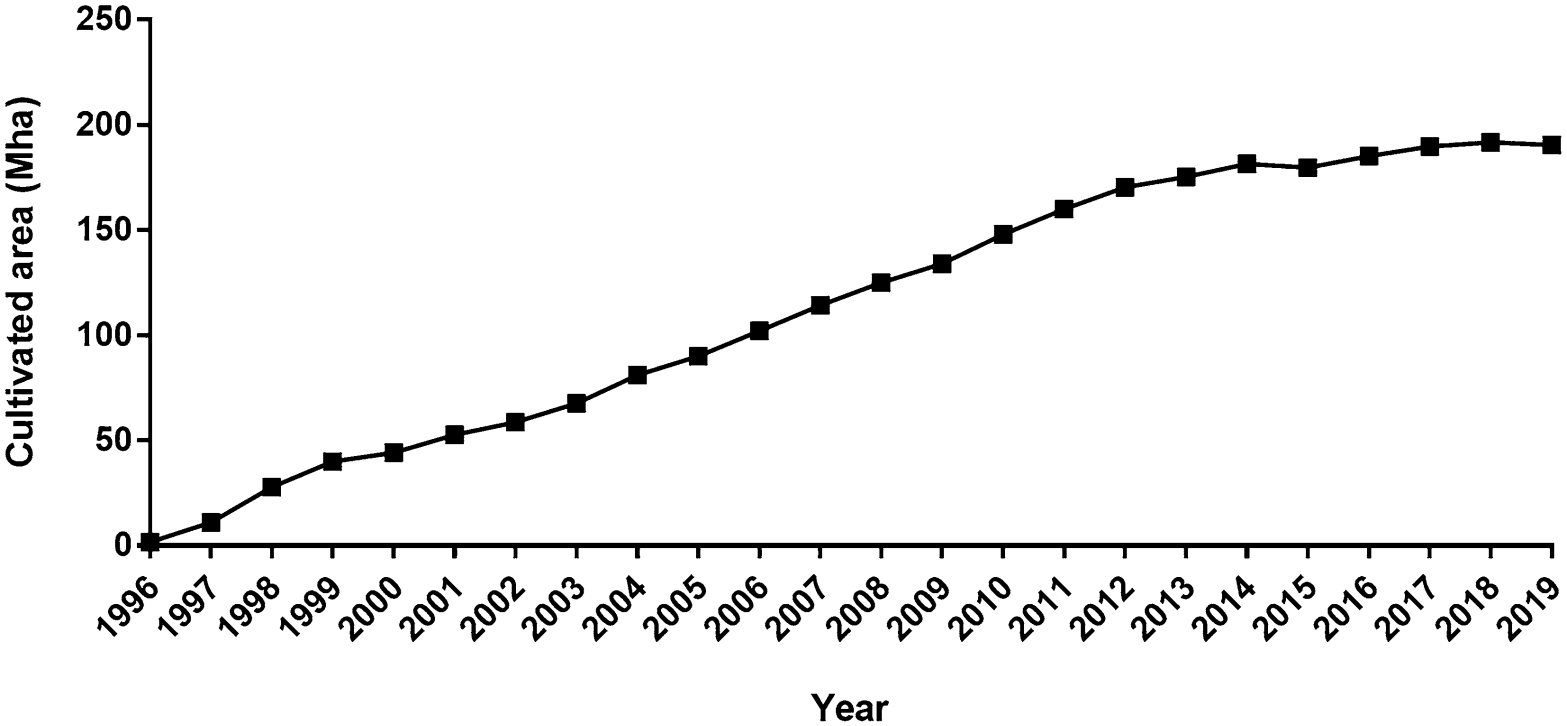
Cultivated area with GM crops worldwide. Plotted from data published in the ISAAA briefs of Global Status of Commercialized Biotech/GM Crops in 2018 and 2019 [6, 8]

Transgenesis has been rapidly implemented in world agriculture in terms of cultivated area. According to the latest reports from the International Service for the Acquisition of Agri-biotech Applications (ISAAA), issued in 2018 and 2019, GM crops have accumulated a total cultivated area of 2.53 billion hectares in 23 years of implementation of this technology (Fig. 1) [6, 8]. In 2019, the last reported year, an area of 190.4 million hectares was cultivated with GMOs in a total of 29 countries, with the Americas being the continent with the largest cultivated area in the world (Table 1).

**Table 1:** Countries with the largest GM crops cultivated area in 2019. The GM crops that have the largest cultivated area in each country are mentioned.

Ranking	Country	Area* (Million ha)	GM crops
1	USA	71.5	Maize, soybean, cotton, alfalfa, canola, sugar beet, potato, papaya, squash, apple
2	Brazil	52.8	Soybean, maize, cotton, sugarcane
3	Argentina	24.0	Soybean, maize, cotton, alfalfa
4	Canada	12.5	Canola, soybean, maize, sugar beet, alfalfa, potato
5	India	11.9	Cotton
6	Paraguay	4.1	Soybean, maize, cotton
7	China	3.2	Cotton, papaya
8	South Africa	2.7	Maize, soybean, cotton
9	Pakistan	2.5	Cotton
10	Bolivia	1.4	Soybean
11	Uruguay	1.2	Soybean, maize
12	Philippines	0.9	Maize
13	Australia	0.6	Cotton, canola, safflower
14	Myanmar	0.3	Cotton
15	Sudan	0.2	Cotton
16	Mexico	0.2	Cotton
17	Spain	0.1	Maize
18	Colombia	0.1	Maize, cotton
19	Vietnam	0.1	Maize
20	Honduras	< 0.1	Maize
21	Chile	< 0.1	Maize, canola
22	Malawi	< 0.1	Cotton
23	Portugal	< 0.1	Maize
24	Indonesia	< 0.1	Sugarcane
25	Bangladesh	< 0.1	Brinjal/Eggplant
26	Nigeria	< 0.1	Cotton
27	Eswatini	< 0.1	Cotton
28	Ethiopia	< 0.1	Cotton
29	Costa Rica	< 0.1	Cotton, pineapple
	Total	190.4	

^*^Rounded to the nearest hundred thousand. Adapted from the ISAAA brief of Global Status of Commercialized Biotech/GM Crops in 2019 [8]

**Fig. 2:**
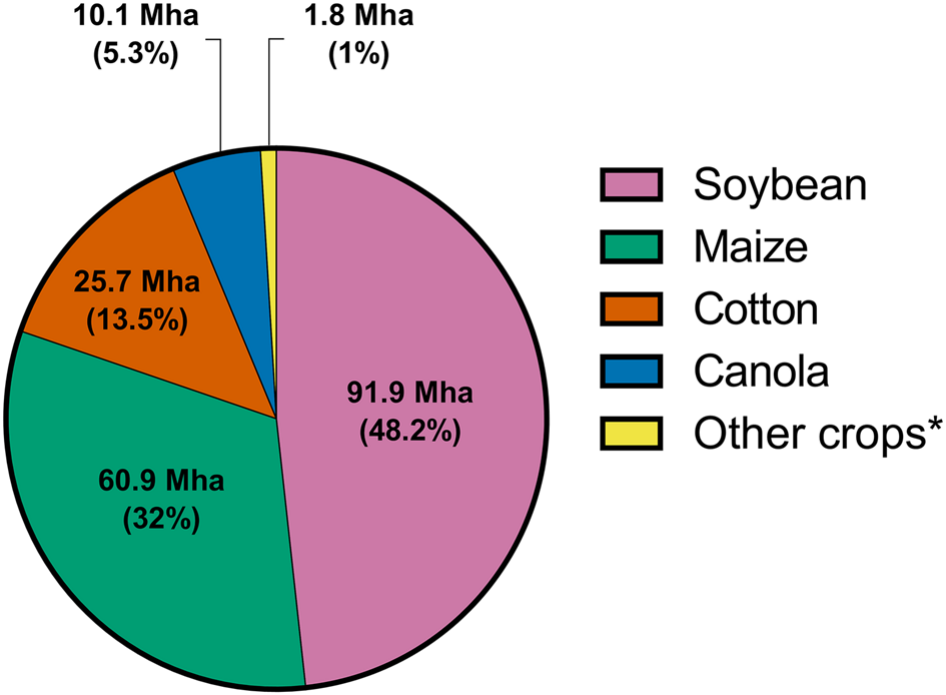
Cultivated area with GM crops reported for 2019. Adoption rate is shown as the percentage of cultivated area with GM crop compared to the total cultivated area for that crop, being GMO or not. [8]. *Other crops: Sugar beet, potato, apple, squash, papaya and eggplant. Adapted from the ISAAA brief of Global Status of Commercialized Biotech/GM Crops in 2019.

The most widely cultivated GM crops are soybean, maize, cotton, and canola, with an area of 188.6 million hectares, which corresponds to 99% of the area cultivated with GMOs worldwide (Fig. 2). About 90% of the area cultivated with GMOs is found in 5 countries (United States, Brazil, Argentina, Canada, and India) (Table 1). Most of the commercially available GM crops have been developed using transgenesis based on recombinant DNA technology, mainly to confer traits such as insect resistance, herbicide tolerance, and tolerance to abiotic stress (> 99% of total commercial traits)[8] (Table 2) or other non-frequent traits related to improved food fortification such as provitamin A biosynthesis in “golden rice” and “golden banana”, or increased starch content in EH92-527–1 potato [9–13]. These transgenic crops have been mainly used for food, livestock and poultry feed, and as ingredients for processed food such as protein extracts, oils and sugar; or for other industries such as ethanol (biofuel) or natural fibre production [14, 15].

**Table 2:** Worldwide GM crops cultivated area by trait

Trait	2017		2018		2019	
	Area (Million ha)	%	Area (Million ha)	%	Area (Million ha)	%
Herbicide tolerance (HT) *	88.7	47	87.5	45	81.5	43
Insect resistance (IR) **	23.3	12	23.7	12	23.8	12
Stacked HT/IR	77.7	41	80.5	42	85.1	45
Others	< 1	< 1	< 1	< 1	< 1	< 1
Total	189.8	100	191.7	100	190.4	100

Adapted and expanded with data from the ISAAA briefs of Global Status of Commercialized Biotech/GM Crops in 2018 and 2019 [6, 8]

^*^Most common traits are tolerance to glyphosate and ammonium glufosinate

^**^Most common trait is Bt, via Cry proteins

### The era beyond transgenesis: genome editing tools

#### New breeding techniques

**Fig. 3:**
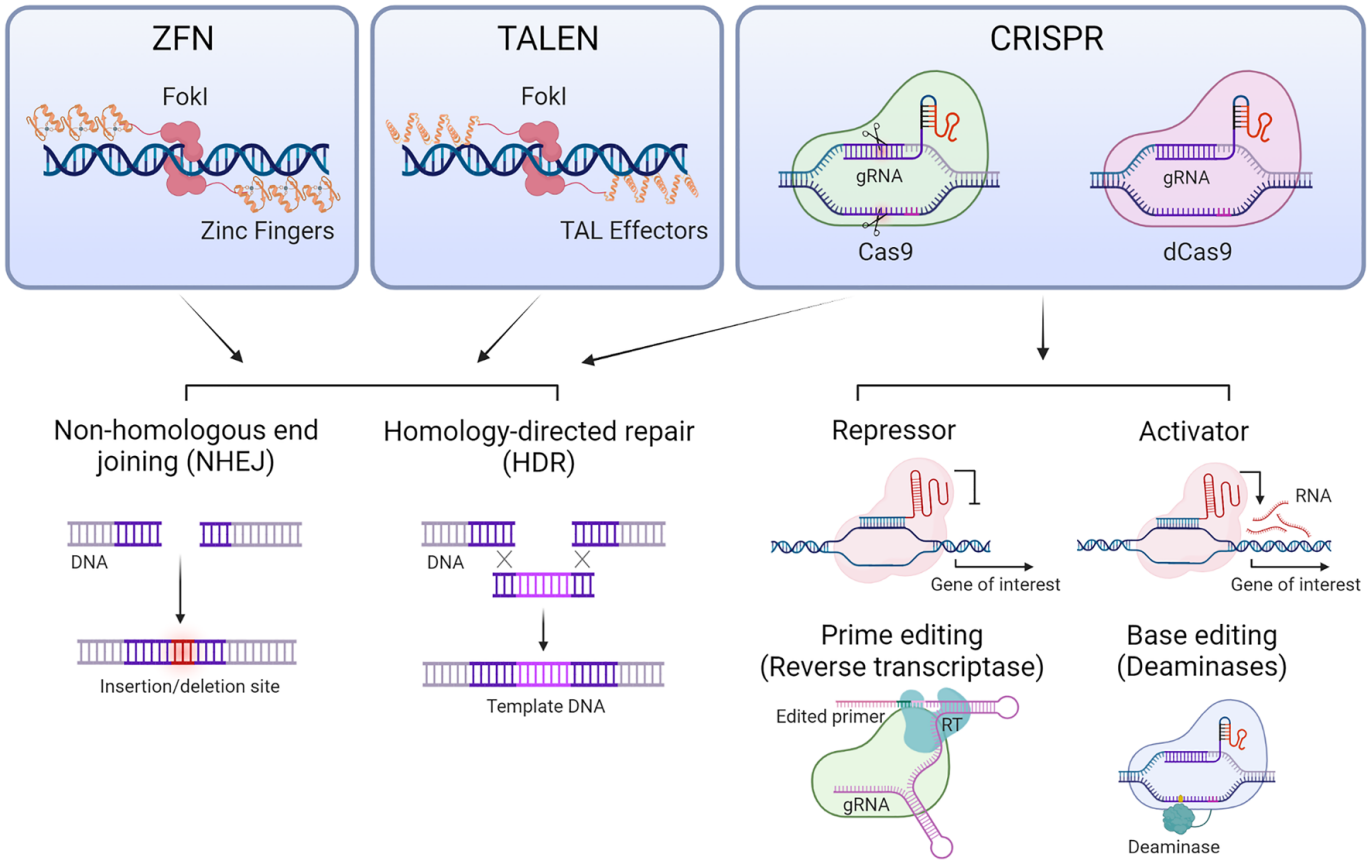
New breeding techniques used for GM crops development. Zinc finger nucleases (ZFN), transcription activator-like effector nucleases (TALEN) and the bacterial system of clustered regularly interspaced short palindromic repeats (CRISPR), employing Fok1 or Cas9 nucleases, are used to target DNA sequences to promote downstream modifications. ZFN, TALEN and Cas9 induce double-strand breaks that are corrected by NHEJ or HDR, modifying the target sequence with deletions or different size insertions. Modified Cas9, such as catalytically null (“dead” Cas9 or dCas9) is used coupled to transcriptional repressor or activators to regulate gene expression. Other forms of modified Cas9, such as coupled to reverse transcriptase (RT) or deaminases, are used to modify target sequence with specific template primers (prime editing) or switch specific bases (base editing).

Along with the process of transgenesis, in the last decade new technologies have been developed that allow editing the genome, or modify its expression, of the target organism in a precise, fast, and relatively cheaper way than other techniques, minted under the acronym "NBT" ("new breeding techniques") (Fig. 3). The genome editing process is based on the use of nucleases able to generate double-strand breaks (DSBs) in specific sequences when guided by proteins or RNA [16]. These breaks are then repaired by the cellular endogenous DNA repair machinery via non-homologous end joining (NHEJ), allowing targeted modifications, such as insertions or deletions, potentially knocking out targeted genes. Moreover, DSBs can also be repaired by homology-directed repair (HDR) using endogenous or delivered template DNA sequence, leading to gene replacement or insertion of sequences of different sizes, from one to many hundreds of nucleotides [17].

The extension, location and downstream effects of these editions will determine the phenotype of the new variety, with novel traits that are, in principle, independent of exogenous gene constructs, thus differentiating them from transgenesis. Nonetheless, the delivery methods used to insert genome editor expression cassettes or ribonucleoprotein complexes represent an obstacle to obtain commercial varieties free of exogenous DNA. This is noteworthy not only for regulatory concerns but also for the acceptance of the final products by consumers [18, 19].

Currently, the three most widely used NBT are zinc finger domain-coupled nucleases (ZFN), transcription activator-like effector nucleases (TALEN) and the bacterial system of clustered regularly interspaced short palindromic repeats (CRISPR) coupled to the nucleases Cas9, Cas12, or Cpf1, among others [16, 17]. ZFN and TALEN systems are based on nucleases, such as Fok1, coupled to tandem zinc finger protein domains or TALE protein repeats, respectively, recognizing specific DNA motifs by protein-DNA interaction. Once this protein-guided DNA interaction occurs, Fok1 nucleases dimerize and perform their enzymatic activity on the double-stranded DNA [16] (Fig. 3). For example, ZFN gene editing has been used on commercially relevant crops to modify endogenous genes involved in different phenotypes such as development in tomato [20], starch metabolism in rice [21], sexual fertility in apple and fig [22], RNA silencing genes in soybean [23] or confer resistance to imidazolinone herbicides in wheat [24]. Potentially commercial traits of interest have been developed using TALEN. For instance, to modify sugar metabolism and improve herbicide tolerance in potato [25, 26], increase oleic acid content in peanut and soybean oil [27, 28], or reduce lignin content and improve saccharification in sugarcane [29, 30]. Early flowering is another trait of interest, allowing to face seasonal and logistic hurdles, and wild cabbage is an example of these research efforts performed by TALEN [31].

#### CRISPR systems

On the other hand, the CRISPR system is based on RNA-guided DNA pairing, taking advantage of the recently growing knowledge of bacterial CRISPR/Cas complex. This RNA–DNA interaction allows sequence specificity design in a more efficient, versatile, and cheaper way than ZFN and TALEN systems [16–18, 32]. Furthermore, the use of tailored Cas complexes has expanded the toolkit for genome editing by incorporating base-switching enzymes, transcription regulators, or adding translational modifications [18]. To date, several variations have been made to the CRISPR/Cas system in order to obtain different modifications on the DNA sequences or regulate gene expression (Fig. 3). Nuclease-inactivated Cas9 (dCas9 for “catalytically dead Cas9”) allows Cas9 targeting DNA sequences guided by a guide RNA (gRNA) without producing strand breaks [33]. The coupling of dCas9 with transcriptional repressors or activators constitutes an adaptable tool to modify gene expression (34). Moreover, fusion of Cas9 with deaminases has been shown to be useful for C-T and A-G base replacement (known as base editors) [33, 35].

In addition to precise base editing, the potential of modified CRISPR/Cas9 systems has been taken into a broader perspective with “prime-editing” tools. In this approach, a reverse transcriptase (RT) is fused to Cas9, and the gRNA is concatenated with template RNA for RT activity. This allows targeting the sequence of interest and editing multiple bases at once without template DNA [36]. In addition to targeting gene expression, CRISPR/Cas systems can also be used to modulate translation by editing upstream open reading frames (uORFs). These uORFs can regulate translation of the primary ORF, as demonstrated in lettuce modified in the LsGGp2 uORF, enhancing vitamin C biosynthesis [37].

CRISPR systems have been experimentally used on most commercial GM crops such as maize, soybean and cotton, along with several other crops, *e.g.*, apple, carrot, orange, raps, lettuce, grapes, pear, strawberry, cucumbers, wheat, rice, and tomato, in addition to ornamental flowers such as *Dendrobium officinale* (orchid), *Ipomoea nil* (morning glory), *Petunia hybrid* (petunia) and *Torenia fournieri* (wishbone flower) [16, 18, 32, 38–40]. The traits obtained by CRISPR also cover a wide range of biotechnological interests such as sugar, fatty acid or pigment metabolism, herbicide tolerance, pathogen resistance, and development modifications, among others.

### Regulatory frameworks

Despite their differences, countries’ regulatory frameworks can be broadly classified as process-oriented or product-oriented [14, 41]. The first determines criteria and regulations according to the methods used to generate new plant varieties, while the second applies criteria based on the new characteristics of a biotechnological event and makes comparisons with their conventional counterparts, both applying a case-by-case evaluation system. Noteworthy, these product- or process-oriented regulatory guides commonly share elements with each other, making legal frameworks difficult to categorize [41, 42]. Parallel to the “regulatory style” of each country, the Cartagena Protocol, signed by more than 140 countries, constitutes an instance of international law with binding legal principles for the countries that have ratified it [7].

Most of the current regulations worldwide have been created to address transgenic-derived crops, which have the largest participation on markets. However, frameworks have been updated in several countries, mainly developed countries, to take genome-edited crops into account. Here we address the current status of regulatory frameworks for GM crops around the globe, in which, for the purpose of this review, we have categorized the main regulatory focus of each country as product- or process- oriented (although it is not a legal classification in any of these countries) (Table 3).

**Table 3:** GMO regulatory frameworks around the word

Region	Regulatory framework	Main regulatory focus*	Additional information**
Americas			
Argentina	Permissive	Process	Declared "Biotech mega-country" by ISAAA
Bolivia	Permissive	Process	Declared "Biotech mega-country" by ISAAA
Brazil	Permissive	Process	Declared "Biotech mega-country" by ISAAA
Canada	Permissive	Product	Unique term of plant with novel traits (PNT). "Declared "Biotech mega-country" by ISAAA"
Chile	Hybrid	Product	Allowed only for seed export and research
Colombia	Permissive	Product	Declared "Biotech mega-country" by ISAAA
Costa Rica	Permissive	Product	NA
Ecuador	Prohibitive	Process	Allowed only for research purposes
Honduras	Permissive	Product	NA
Mexico	Permissive	Product	Declared "Biotech mega-country" by ISAAA
Paraguay	Permissive	Product	Declared "Biotech mega-country" by ISAAA
Peru	Prohibitive	Process	Allowed only for research purposes
Uruguay	Permissive	Product	Declared "Biotech mega-country" by ISAAA
USA	Permissive	Product	Declared "Biotech mega-country" by ISAAA
Venezuela	Prohibitive	Process	Total moratorium
Europe			
European Union	Restrictive	Process	Only cultivated in Spain and Portugal
Africa			
Burkina Faso	Permissive	Process	No current commercial production
Egypt	Prohibitive	Process	Allowed only for research purposes
Eswatini	Permissive	Process	NA
Ethiopia	Permissive	Product	NA
Ghana	Prohibitive	Process	Allowed only for research purposes
Kenya	Permissive	Product	NA
Malawi	Permissive	Product	NA
Nigeria	Permissive	Product	NA
South Africa	Permissive	Process	Declared "Biotech mega-country" by ISAAA
Sudan	Permissive	Product	Declared "Biotech mega-country" by ISAAA
Uganda	Prohibitive	Process	Allowed only for research purposes
Asia and Oceania			
Australia	Permissive	Process	Declared "Biotech mega-country" by ISAAA
Bangladesh	Permissive	Product	NA
China	Permissive	Process	Declared "Biotech mega-country" by ISAAA
India	Permissive	Process	Declared "Biotech mega-country" by ISAAA
Indonesia	Permissive	Product	NA
Japan	Hybrid	Product	Allowed only for ornamental plants and research
Myanmar	Hybrid	Process	Allowed only for non-food crops. Declared "Biotech mega-country" by ISAAA
New Zealand	Restrictive	Process	Several socio-cultural restrictions
Pakistan	Permissive	Process	Declared "Biotech mega-country" by ISAAA
Philippines	Permissive	Product	Declared "Biotech mega-country" by ISAAA
Vietnam	Permissive	Product	Declared "Biotech mega-country" by ISAAA

^*^ Product- or process-orientation is used as a practical guideline and is not a legal classification

^**^ Biotech mega-country is defined by ISAAA as growing more than 50.000 hectares of GM crops [8]

*NA* No additional information

#### Americas

##### Latin America

In Latin America there are different types of regulation, from very permissive to moratorium. The lack of consensus is a characteristic of the region, where countries with similar regulations do not coordinate or share information on seeding requests. The “biotechnological mega-countries” (a term coined by ISAAA for countries growing more than 50.000 Ha of GM crops) in the region, being Argentina, Brazil, Bolivia, Colombia, Mexico, Paraguay and Uruguay, have regulations that allow the cultivation and/or trade of GMO, rendering them as important players in the global market. Other Latin America countries with planted GMOs such Costa Rica and Honduras, have similar regulations compared to the biotechnological mega-countries of the region. Chile has a unique regulatory framework where GMO crops can be planted for seed production and export, and research purposes but not for domestic food or feed uses [43, 44]. Despite the regulation similarities within these countries, their definitions and lists of approved events are not synchronized, which delays the application of events throughout the region and weakens regional trade [14].

Therefore, at the XXXIV Extraordinary Meeting of the Southern Agricultural Council (CAS), held in 2017, the Ministers of Agriculture of Brazil, Chile, Paraguay and Uruguay, and the Secretary of Livestock, Agriculture and Fisheries of Argentina, declared it necessary to promote activities of regional cooperation and exchange of information for the approval of GMOs and to train "experts in new technologies" (related to NBT). The common characteristic in the regional regulations relies in evaluating food biosafety and field release (environmental and biodiversity) in a case-by-case and product-oriented manner, according to the institutions mandated for such purposes in each country [14].

On the prohibitive side of Latin American region, Ecuador, Peru, and Venezuela do not allow the commercial cultivation of GMOs. Ecuador, whose 2008 constitution defines the country as "free of transgenic crops and seeds", has made its position more flexible and allows the use of GM seeds for research purposes, through the Organic Law of Agrobiodiversity, Seeds and Promotion of the Sustainable Agriculture, decreed in 2017. In the case of Venezuela, the Venezuelan Seed Law, decreed in 2015, prohibits all GM crops in its territory.

In Peru, a transition towards a prohibitive policy has been observed. In 1999, through Law 27104, the regulation of GMOs was established, managing and controlling their confined use and release; in addition to regulating its introduction, commercialization, research, transportation, and storage, among others. It even decreed a law for the labelling of foods made with ingredients that contain GMOs (Law 29888). However, through the enactment of Law 29811, in 2011, a moratorium on the entry and production of GMOs in Peruvian territory was established for ten years, emphasizing the need to assess risks, protect biodiversity, and generate a new regulatory framework. This moratorium excludes GMOs cultivated for research purposes and in January 2021 the Peruvian Congress enacted an extension of the moratorium for fifteen more years from the end of the first ten-year period (Law 31111).

##### USA and Canada

The United States of America (USA) and Canada share a common regulatory style, considering the new GM plant varieties as conventional based on case-by-case biosafety analysis. This permissive style has allowed these countries to use their previous legislation to adapt it to the evaluation of GMOs [14]. Following this line, the USA, despite being the main producer of GM crops in the world, does not have federal legislation as a general framework to regulate GMOs. Depending on whether the purpose of the GM product is for human, animal and/or environmental use, its authorization and regulation fall under the standards of the Food and Drug Administration (FDA); the Animal and Plant Health Inspection Service (APHIS); or the Department of Agriculture (USDA) and/or the USA Environmental Protection Agency (EPA), respectively.

The case of Canada is unique in the world, considering a new term in its regulatory framework: “plant with novel traits” (PNT). In the Canadian regulatory framework, a new plant variety is considered a PNT if it meets certain differentiating criteria with its conventional counterpart, regardless of the methodology used to generate it, be it transgenesis, conventional breeding or NBT. Therefore, a new plant variety can be considered a PNT in Canada while being considered a GMO for the rest of the world. Despite the broad definition criteria, Canada has a biosafety evaluation system focused on toxicity, allergenicity, impact on field release and even impacts on organisms other than the PNT, through the Canadian Food Inspection Agency (CFIA) [11].

#### Europe

The regulations of the European Union (EU) have been classified as restrictive since they determine high biosafety standards for human and animal consumption, environmental impact and consumer interests, as stated in the first article of Regulation 1829/2003 EU. This standard gives much of the responsibility on the applicant to demonstrate the safety of the GM product and to monitor its cultivation or use as food. In addition, the EU regulation provides a framework for citizen participation by making public the Authority's opinions regarding new requests. Citizens can send their comments to the evaluation committee within a period of thirty days, through article 6 number 7 of the aforementioned Regulation. Such have been the levels of control in the EU in terms of applications to cultivate GMOs that in more than two decades only two biotechnological events have been approved for cultivation and in the last years only one is cultivated in Spain and Portugal (insect-resistant corn, MON810) [8].

Despite this, the EU is one of the main importers of GMOs for human consumption, being mainly soybeans and its derivatives (90–95% GMOs of total imports), maize (20–25% GMOs of total imports) and canola (25% GMOs of total imports) [8]. In addition to seeding restrictions, it has regulations on traceability and labelling of GMOs. The general standard of the EU regulatory style is based on the definition of process-oriented GMOs, defining a GMO in article 2 of Directive 2001/18/EC, as “if the method of genetic modification is carried out in such a way that does not occur by natural crossing and/or recombination”. This definition does not take into account the type of modification, be it gene insertion, regulatory sequences, specific nucleotide changes, etc. Therefore, it does not discriminate the type of methodology used to generate a GMO. On the other hand, this definition also includes conventional plant breeding, on which cases it has an allowing “historical” criterion.

#### Africa

The African continent has been slowly adopting GM crops with different regulations between countries, similar to Latin America. Africa is home to some of the countries with the largest area planted with GM crops in the world (South Africa, Sudan, Nigeria, Eswatini and Ethiopia) (Table 1), in addition to Malawi and Kenya. With regard to the cultivated plant varieties, South Africa has cultivated maize, soybeans and cotton, while other countries cultivate mainly IR/Bt cotton [8], for a total of 2.9 million hectares of GM crops in 2019.

South Africa was the first African country to regulate GM crops through the Genetically Modified Organisms Act No. 15 of 1997, while other countries began to regulate this technology since the early 2000’s (Kenya and Malawi) or the last decade (Egypt, Ethiopia, Eswatini, Ghana, Nigeria, Sudan, Burkina Faso and Uganda). Furthermore, Egypt, Ghana and Uganda do not allow GM crops cultivation for commercial purposes [14]. Burkina Faso has been producing Bt cotton since 2008 but stopped its production in 2016 due to quality concerns. Its regulation allows cultivation of GM crops, but there is currently no commercial production [45]. Although Egypt was a pioneering African country in developing and planting GM maize in 2008, GM cultivation was banned four years later due to a lack of biosafety laws [46].

#### Asia and Oceania

Asia is the main source of GM cotton, with India being the country with the largest cultivated area (11.9 million hectares of Bt cotton in 2019) [8]. Despite the approval of Bt cotton in India in 2002, several other food and non-food GM crops are not allowed and have been planted illegally since then, such as virus-resistant papaya, Bt brinjal/eggplant and IR/HT cotton [47]. Like India, Pakistan and China are also ones of the main producers of Bt cotton [8]. Beyond the domestic and export production of GM crops, China has led the research and development of GMOs obtained by NBT, being the main source of published articles and patent applications in this regard [48, 49]. The Ministry of Agriculture and Rural Affairs is the institution responsible for new approvals and demands strict field and environmental assessments for new events, delaying the process from development to commercialization. This marks a difference with the USA and Canadian regulatory frameworks, that allow faster track for the application of new events [14].

Philippines is one of the key players in the market of GMOs in Southeast Asia, being a leading producer of GM maize in the region, and also an important commercial target for GM rice that harbours enzymes for the biosynthesis of the vitamin A precursor (golden rice), which is produced mainly in China [9]. Like Philippines, Indonesia also has a product-oriented regulation with the difference of a smaller production of GMO limited to sugarcane [8]. Similar to these cases, Vietnam and Bangladesh, in Asia mainland, also have a permissive regulatory style regarding GMOs but only one species is the main focus of production being maize and brinjal/eggplant, respectively [8].

In the Pacific region, New Zealand has a strict regulatory framework that takes Māori culture into consideration, prohibiting crops that may alter traditions, sites, flora, and fauna [50]. This has led to no GM crops being cultivated commercially in the country. Moreover, this regulatory framework also considers new plant varieties developed by NBT through the regulation of GMOs [41].

Japan and Australia allow the cultivation of GM crops but with different regulatory approaches. Japan leads in GM crops approvals behind the USA, but its strict confined field trials and environmental risk assessments have not allowed commercial production of GMOs for food or feed, but only for ornamental blue rose flower [51]. On the other hand, Australia has allowed commercial production of GM crops, being a major producer of cotton, canola, and safflower (ranked 13th in area cultivated with GMOs in 2019) (Table 1) [8]. Despite their different approaches to commercial cultivation of GMOs, Japan and Australia share common criteria for evaluating and defining new plant varieties developed by NBT, considering unguided repair of site-directed nuclease activity (SDN-1) organisms as non-GMO [52, 53].

### Beyond GM crops

#### GM microorganisms

Agriculture has been the activity with the most extensive research, development, and application of GMOs. However, several other fields have been taking advantage of this technology. Closely related to crops, the use of yeast has been a historical tool for the production of bread and alcoholic beverages (such as wine and beer). Furthermore, due to the extensive knowledge of yeast genetics and cell biology, the biotechnological application of yeasts, as well as other fungal species, has rapidly evolved and spans various industries, such as biofuel production, medical applications, and alcoholic beverages itself. For example, genetic modification of yeast strains has been experimentally tested to modulate ethanol yields [54, 55].

Although the use of GM yeasts in industrial applications such as bioethanol and pharmaceutical production is not a problem (the commercialization of recombinant insulin is an example of this), the use of GM yeasts for food production has faced the same problems associated with GM plants, *i.e.*, legal restrictions and consumer rejection, which lead to the limited commercial success that recombinant yeasts have had in the food industry [56, 57]. For example, in the wine industry, there are only two commercialized GM strains: one for better metabolization of urea [58] and other for simultaneous alcoholic and malolactic fermentation [59]. Most commercialized wine yeast strains have resulted from the selection of strains naturally present in different ecosystems [60–62], followed by hybridization [63–65], and, in recent years, from breeding programs (similar to those made in plants and animals) [57, 66, 67].

All the aforementioned aspects are relevant not only for the use of yeast but also other microorganisms for food production, *e.g.*, lactic acid bacteria. And because NBT can also be applied for genome modification of microorganisms, the impact that these technologies could have in regulations worldwide will also impact the development and commercialization of new strains of microorganisms with enhanced characteristics.

#### Biomedical applications

Biomedical sciences have been systematically exploiting genetic modification for new therapeutic approaches since the 90's. The practical potential of these approaches comes from complementary fields in continuous development: the design and optimization of in vivo oligonucleotide-based therapies, engineering of viral vectors for gene therapy and the introduction of gene-edited cells generated ex vivo into patients to treat certain conditions, especially blood-related diseases [68–70]. Importantly, these gene editing techniques have been employed to modify coding or non-coding regulatory sequences and also epigenetic modulators of gene expression (71, 72). Noteworthy, the engineered viral vectors and the genetically modified cells can be considered GMOs or products of them, depending on the methodology used.

Despite increasing knowledge and proof-of-concept studies, only a few gene-editing therapies have been approved by FDA and are currently available to patients [69, 73]. Most of these therapies are based on chimeric antigen receptor T cells (CAR-T cells), modifying T lymphocytes ex vivo with viral vectors to infuse them back into the patient’s bloodstream to treat multiple myeloma or B-cell lymphoma. Trade names for these FDA-approved CAR-T cells therapies are Abecma, Breyanzi, Carvykti, Kymriah, Tecartus, Yescarta. Besides the ex vivo approach, Imlygic is the only case of local administration of viral particles to transduce cancer cells, leading to oncolysis for melanoma treatment. Luxturna and Zolgensma are adeno-associated virus (AAV) gene therapies for *RPE65* mutation-associated retinal dystrophy and spinal muscular atrophy (SMA), replacing the dysfunctional alleles of the *RPE65* or *SMN1* gene, respectively, with their functional copies [74, 75]. These two gene-replacing AAV therapies constitute the only approved cases for gene editing of the nervous system.

Controversially, the patient’s somatic cells transduced in gene therapy administration can also be considered as GMOs, since they meet the definition of the Cartagena protocol, as long as they harbour a new combination of genetic material through the use of modern biotechnology. For example, Luxturna and Zolgensma viral vectors replace the dysfunctional alleles of the *RPE65* and *SMN1* genes in retina or central nervous system nerve cells, respectively. This results in genetically modified somatic cells. How will GMO regulation take these events into account? This question is still open for debate, as regulatory frameworks keep pace with new technologies and applications.

Beside genome edition approach to develop therapeutic interventions, targeting gene expression has also been tested by meanings of RNA-based therapies [76]. Contrary to the case of some viruses, DNA but no RNA is considered as the genetic material in humans and, thus, RNA use and/or modification would not be regarded as LMOs by Cartagena protocol [77]. Nevertheless, nucleic acid therapies based on RNA have been proved useful to treat several diseases and their regulation could fall under the terms of genetic modification if the case arises. These therapies include vaccines, being COVID-19 messenger RNA (mRNA)-based ones the most widespread employed up to date [78, 79]. One of the main advantages of RNA therapy is the reduced genotoxicity due to lack of integration into the genome [76]. Moreover, due to the diverse roles of RNA molecules in cell biology, including modulation of transcription, mRNA processing, translation and protein homeostasis, is possible to target specific metabolic pathways without carrying the modification into daughter cells [76, 80–82]. RNA-based therapies have been approved by FDA to treat several diseases, such as atherosclerotic cardiovascular disease (ASCVD) and hypercholesterolemia [83, 84], SMA [85], Duchenne muscular dystrophy [86, 87], hereditary transthyretin-mediated amyloidosis (hATTR) [88], hepatic porphyria [89] or neovascular age-related macular degeneration [90]. These therapies relay on antisense oligonucleotide, small interfering RNA (siRNA) or modified RNA (aptamers) tools [76].

Not only human cells are the main target for gene editing or gene expression modification in pathological contexts. The use of biomaterials in medicine has opened new avenues for GMOs and/or their products in biomedical treatments. Spanning from tissue engineering, drug delivery, organ transplantation, artificial organs, dental implants, bone replacement to prosthetics, among others, biomaterials serve as a functional platform to couple GMOs to human physiology. Stratagraft and Maci are FDA-approved cellular therapies acting as scaffolds for tissue regeneration indicated for knee cartilage defects or deep partial-thickness thermal burns, respectively. Despite not being genetically modified, these decellularized collagen scaffolds open the way for existing and developing “functional” biomaterials that express recombinant proteins such as growth factors, immune modulators or extracellular matrix components [91–93]. Following this line, functional photosynthetic scaffolds for dermal regeneration have been tested using *Synechococcus sp.* transgenic cyanobacteria that synthetize hyaluronic acid or modified *Chlamydomonas reinhardtii* microalgae that expresses the vascular endothelial growth factor (VEGF) [92, 93].

#### GMOs for climate change challenge

Notwithstanding the potential of GMOs to face big challenges in human activities, regulatory frameworks and public opinion continue to play a critical role in their development and implementation. Such is the case of climate change solutions based on GMOs. It has been proposed that biotech crops can reduce the greenhouse gases (GHG) emission by means of optimizing land-use, increasing yields, and decreasing the chemical, energy and transport resources involved in agricultural production [94]. Herbicide and insect resistant traits have allowed reduced levels of pesticide used worldwide estimated to an extent of 8.3% compared to the amount needed on the same area planted with conventional counterpart crops [95]. This have led to reduced, and even remove, tillage between agricultural cycles because farmers no longer need to remove weeds mechanically neither separate pathogen-infected plants [95]. Due to this continuous use of land for crop growth, there is more plant mass available to change atmospheric CO_2_ fluxes towards the soil in a phenomenon termed carbon sequestration [96]. Moreover, insect resistant traits have reduced the need for insecticide spraying, decreasing the fuel consumption associated with this process worldwide. In top of that, some authors argue that GM crops require less agricultural surface to be produced, also decreasing the fuel demand for machinery associated with larger farm area [94, 95]. It has been estimated that, depending on the region, cultivation of maize, soybean or rotation of both, have a carbon sequestration between 102 and 250 kg of carbon per hectare per year [95].

European geographical conditions are advantageous for growing the most commercialized GM crops. It has recently been estimated that GMOs adoption in the EU will increase yields and lower pesticide utilization [94]. Importantly, the EU imports more than 45 million tons of maize and soybean, for food and feed, from the Americas (mainly USA, Argentina and Brazil). Higher yields and increased local production due to hypothetical adoption of GM crops in the EU will reduce imports and therefore the environmental impact worldwide. This scenario could lead to a reduction in GHG emissions by 33 million tons of CO_2_ equivalents per year [94]. However, as stated above, Europe is the most reluctant region to GMOs adoption due to its strict regulatory framework and overall consumer rejection. As long as these legal and sociological features hold their positions, little progress will be made not only in assessing GM crops potential to tackle climate change, but also in scientific research for European crops breeding and global solutions. Nevertheless, a future turn towards uses of modern biotechnology could be expected as the presence of GM ingredients in food and drinks and gene editing technology are not even at the top three main concerns regarding food security, according to the last Eurobarometer survey assessing food security perception [97, 98].

In addition to GHG emissions reduction and crop yield and nutrient content optimization, plant adaptation to the changing environment is one of the main concerns in climate change context. Besides conventional breeding, genetic modification has been tested to enhance plant resistance to higher global temperatures and lower water availability. In this scenario, the drought-tolerance trait has become an attractive research focus for crop development [99, 100]. Soybean and wheat, two of the most consumed crops, have been modified to express sunflower Hahb-4 transcription factor related to water stress responses [101]. These transgenic crops (termed HB4 crops) are currently commercially available and do not differ in nutritional content compared to their non-transgenic counterparts [102–104]. Under field conditions, HB4 soybean has increased seed yield and water use efficiency in dry environments compared to non-transgenic crops [105]. Experience in the USA has shown that one of the few drought-tolerant commercial maize led to increased yields in water-limited environments compared to conventional hybrids in the same regions, with yield differences ranging from 1 to 9.7% [106, 107]. The understanding of water management and root systems in plant biology is a key aspect for the development of this trait [100]. In fact, modifying rice root architecture-related locus *Dro1*, increased root depth and provided better yields under water-limited in vitro or field environments [108, 109]. Another relevant path to stress resistance and drought tolerance is abscisic acid (ABA) hormone signaling, being a potential target for genetic modification in order to obtain new varieties [100]. Transgenic canola harboring antisense construct against farnesyltransferase (ERA1), an ABA signaling down-regulator factor, is able to increase seed yields under water-limited field conditions during flowering time [110]. Indeed, complementary approaches modifying root systems, ABA signaling and early-flowering strategies could be useful to cope with warm seasons, avoiding exposition to heat and reduced water levels in drought risk regions [99, 100, 110–112].

## Conclusions

The development of new genetic editing strategies and technologies such as NBT has brought opportunities to face critical challenges in different aspects of human life. From meeting the needs for food and feed to development of industries and new therapeutic approaches, the enormous potential of LMOs could be a game-changing tool to thrive in a rapidly changing world. Updating, understanding and discussing this scientific knowledge will have a profound impact on regulatory frameworks across the world as seen in the evolution of different legal styles that has been constructed over the years. Noteworthy, the comparison of these frameworks shows that several cultural and local aspects, such as environmental or economic factors, are as important as technology development to rise up to these challenges.

## Data Availability

Not applicable.

## Abbreviations

AAV: Adeno-associated virus
ABA: Abscisic acid
CAR-T: Chimeric antigen receptor T
CRISPR: Clustered regularly interspaced short palindromic repeats
DSB: Double-strand break
DNA: Deoxyribonucleic acid
GHG: Greenhouse gases
GM: Genetically modified
GMO: Genetically modified organism
HDR: Homology-directed repair
HT: Herbicide tolerance
IR: Insect resistance
NBT: New breeding techniques
NHEJ: Non-homologous end joining
LMO: Living modified organism
ORF: Open reading frame
PNT: Plant with novel traits
RNA: Deoxyribonucleic acid
RT: Reverse transcriptase
SMA: Spinal muscular atrophy
TALE: Transcription activator-like effector
TALEN: Transcription activator-like effector nuclease
VEGF: Vascular endothelial growth factor
ZFN: Zinc finger domain-coupled nucleases
